# 

**Published:** 1860-01

**Authors:** 


### CONTENTS.

Essay on Crown Cavities in Molar Teeth. By GEO. L. PAINE .......... 297
The Physiology and Pathology of the Mouth .......... 300
Impressions. By Dr. M. M. OLDHAM .......... 305
Professional Advertising. By C. C. DILLS .......... 308
Dental Case .......... 315
Serum in Pulp Cavities .......... 317
Correspondence .......... 318
Thoughts for Dentists .......... 318
The Human Teeth .......... 320
Merriam's Tongue Holder .......... 323
Selections .......... 324
Adaptation of the Human Eye to Distances .......... 324
Case of Removal of Half of the Lower Jaw. By J. MASON WARREN, M. D. .......... 326
Separating Teeth by Mechanical Pressure. By J. CANNING ALLEN .......... 328
Finishing Plate-Work. By J. L. SUEESSEROTT .......... 330
Exposed Nerves. By G. EZRA M'KOWN .......... 332
"Fees for Professional Services," .......... 333
Cases of Extirpation of the Parotid Gland and other Glandular Tumors .......... 335
Editorial .......... 340
Warranting Dental Operations .......... 340
Declaration of Independence .......... 341
Vulcanized Rubber .......... 343

